# Risk factors for actinic cheilitis: A meta-analysis

**DOI:** 10.34172/joddd.2021.047

**Published:** 2021-12-05

**Authors:** Alberto Rodriguez-Archilla, Amna Irfan-Bhatti

**Affiliations:** Department of Stomatology, Oral Medicine Unit. Faculty of Dentistry, University of Granada, Granada, Spain

**Keywords:** Cheilitis, Precancerous conditions, Risk factors, Sunlight

## Abstract

**Background.** Actinic cheilitis (AC) is a potentially malignant disorder characterized by chronic lip inflammation, especially the lower lip, associated with accumulative exposure to solar radiation. The present study aimed to assess the possible risk factors related to AC.

**Methods.** A search for studies on AC risk factors was conducted in the following databases: PubMed (MEDLINE, Cochrane Library), Web of Science (WoS), and Google Scholar. For dichotomous outcomes, the estimates of the effects of intervention were expressed as odds ratios (ORs) using Mantel-Haenszel (M-H) method, and for continuous outcomes, the estimates of the effects of intervention were expressed as mean difference (MD) using the inverse variance (IV) method, both with 95% confidence intervals.

**Results.** Twelve studies were considered in this meta-analysis. The factors from the highest to lowest risk of AC were having a low skin phototype (OR: 3.30), age >50 years (OR: 3.01), having high sun exposure, cumulative throughout life (OR: 2.13) as daily (OR: 2.00), being male (OR: 1.78), and being a drinker (OR: 1.56) or smoker (OR: 1.32). However, the use of sunscreen creams and caps/hats to protect against the sun were factors with no significant influence on the AC risk.

**Conclusion.** Chronic sun exposure in subjects with low skin phototypes is the main risk factor for AC.

## Introduction

Actinic cheilitis (AC) is a potentially malignant disorder characterized by chronic inflammation of the lip, especially the lower lip, which is associated with chronic and cumulative exposure to ultraviolet (UV) radiation. The prevalence of AC ranges between 0.4% and 2.4% of the general population, although in susceptible groups with outdoor activities, it might reach 43.2%.^1^ Clinically, AC is characterized by areas of erythema, atrophy, edema, and desquamation that can evolve into erosions or white patches in advanced cases. A diffuse border between lip skin and labial semimucosa might also be seen.^2^ Histologically, AC exhibits atypia and loss of polarity of the keratinocytes, chronic inflammatory infiltrate, and elastosis in the connective tissue due to basophilic degeneration of the extracellular matrix (solar elastosis). In advanced cases, dysplastic changes might be observed.^3^

The main risk factor for AC is chronic and cumulative exposure to solar radiation. Other related risk factors include long outdoor work and activities, smoking habits, lighter skin tones, and immunosuppression. Fair-skinned subjects (low skin phototypes) are more susceptible to sun damage and more prone to developing AC. The process of malignant transformation of AC into lip carcinoma is usually very slow (1–30 years), with a transformation rate of 3‒16.9%. Therefore, early diagnosis and treatment of AC are of great importance to prevent its progression to lip carcinoma.^4^

The present study aimed to assess the potential risk factors related to AC.

## Methods

**Figure 1:**
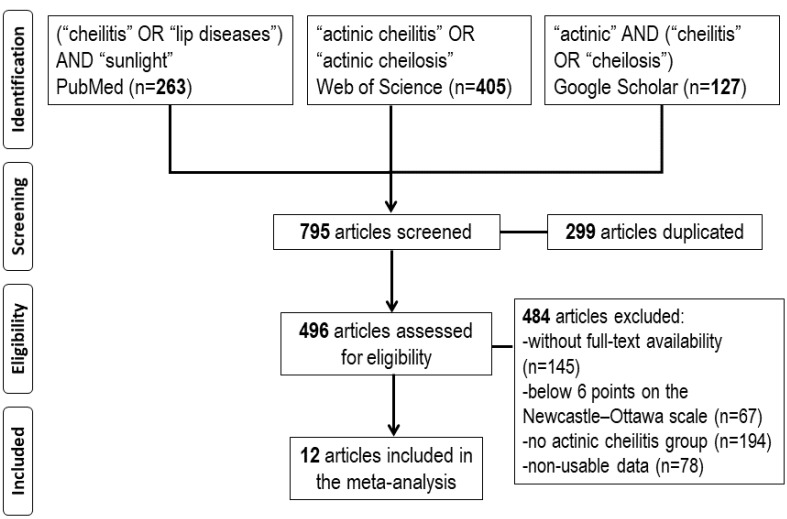
Study flow diagram.

A search for studies on AC risk factors was conducted in the databases PubMed (MEDLINE, Cochrane Library), Web of Science (WoS), and Google Scholar (Google Scholar). A combination of Medical Subjects Headings (MeSH) and free-text terms were used as search strategies. The search terms were the following: (“cheilitis” [MeSH Terms] OR “lip diseases” [MeSH Terms]) AND “sunlight” [MeSH Terms]; (“actinic cheilitis” OR “actinic cheilosis”); allintitle: “actinic” AND (“cheilitis” OR “cheilosis”). After this initial search, 795 articles were found (263 in PubMed, 405 in WoS, and 127 in Google Scholar) from 1952 to 2020, 299 of which were duplicates, leaving 496 eligible articles. The two authors jointly agreed upon the study selection process for the meta-analysis. The exclusion criteria were: a) articles without full-text availability (n = 145), b) articles with a score of <6 stars out of a maximum of 9 on the Newcastle-Ottawa methodological quality assessment scale (n = 67),^5^ c) articles without a control group (n = 194), and d) studies with non-usable data (n = 78). After applying these criteria, 12 studies were included in this meta-analysis (Figure 1).

### Statistical Analysis

The data were processed with the RevMan 5.4 software for meta-analysis (The Cochrane Collaboration, Oxford, UK). For dichotomous outcomes, the estimates of effects of the intervention were expressed as odds ratios (ORs) using Mantel-Haenszel (M-H) method. For continuous outcomes, the estimates of the effects of intervention were expressed as mean difference (MD) using the inverse variance (IV) method, both with 95% confidence intervals. Heterogeneity was determined according to the *P* values and the Higgins statistic (I^2^). A random-effects model was applied when the heterogeneity was high (I^2^ > 50%). A *P* value < 0.05 was considered statistically significant.

## Results

Table 1 presents the main descriptive characteristics and the methodological quality according to the NOS scale of the twelve studies included in this meta-analysis.^6-17^

**Table 1:** Descriptive characteristics of the 12 studies included in the meta-analysis

**Study**	**Year**	**Country**	**Study populations** **n (gender)**	**Parameters analyzed**	**NOS**
Junqueira et al^6^	2011	Brazil	80 AC (12M,68F) 122 noAC (24M,98F)	age groups, gender, skin phototype, sun exposure, smoking, drinking, schooling.	7
Martins-Filho et al^7^	2011	Brazil	40 AC (25M,15F) 200 noAC (66M,134F)	age groups, gender, skin phototype, sun exposure, smoking.	7
de Souza Lucena et al^8^	2012	Brazil	75 AC (60M,15F) 89 noAC (66M,23F)	age groups, gender, skin phototype, sun exposure, smoking, drinking, photoprotection, outdoor jobs.	7
de Souza Lucena et al^9^	2012	Brazil	57 AC (49M,8F) 304 noAC (214M,90F)	age groups, gender, skin phototype, sun exposure, smoking, drinking, photoprotection, outdoor jobs.	7
Orozco et al^10^	2013	Brazil	25 AC (16M,9F) 126 noAC (68M,58F)	gender, skin phototype, sun exposure, smoking, photoprotection.	6
Barreiros et al^11^	2014	Brazil	4 AC (2M,2F) 196 noAC (74M,122F)	age groups, gender, skin phototype, sun exposure, smoking, drinking.	6
de Oliveira Ribeiro et al^12^	2014	Brazil	24 AC (15M,9F) 186 noAC (99M,87F)	age groups, gender, skin phototype, sun exposure, smoking, photoprotection.	6
Ferreira et al^13^	2016	Brazil	138 AC (111M,27F) 272 noAC (157M,115F)	age groups, gender, skin phototype, sun exposure, smoking, drinking, schooling, photoprotection.	7
Rios et al^14^	2017	Chile	70 AC (70M,0F) 110 noAC (107M,3F)	age groups, gender, skin phototype, sun exposure, smoking, drinking, photoprotection.	7
Rodriguez-Blanco et al^15^	2018	Spain	410 AC (183M,227F) 829 noAC (324M,505F)	gender, skin phototype, sun exposure, smoking, outdoor jobs.	7
Santos et al^16^	2018	Brazil	78 AC (78M,0F) 123 noAC (123,0F)	skin phototype, sun exposure, photoprotection.	7
Moreira et al^17^	2020	Brazil	83 AC (58M,25F) 157 noAC (84M,73F)	age groups, gender, skin phototype, sun exposure, schooling, photoprotection.	7

AC: patients with actinic cheilitis; noAC: subjects without actinic cheilitis; M: male; F: female; NOS: Newcastle-Ottawa quality scale.

A total of 3798 individuals, 1084 patients (28.6%) with AC and 2714 (71.4%) without this lip lesion were recorded in these studies. By gender, 679 males (62.6%) and 405 females (37.4%) were found in the group of AC patients, with 1406 males (51.8%) and 1308 females (48.2%) in non-AC subjects. Considering the Newcastle-Ottawa (NOS) quality scale,^5^ only articles with low to moderate risk of bias (≥6 stars) were included in the present study. Nine articles (75.0%) achieved 7 points on the NOS scale, while the other three (25.0%) had 6 points.

The analyses of different AC risk factors are presented in Table 2.

Seven studies^5-11^ analyzed whether being older or younger than 50 could influence the risk of developing AC. Patients >50 years of age were 3.01 times more likely to develop AC with a highly significant statistical relationship (OR = 3.01; 95% CI: 1.88‒4.80, *P* < 0.001). Four studies^6,9,12,13^ considered the mean age of AC patients and controls, reporting a mean age of 8.13 years higher in AC patients. Statistical analysis revealed a highly significant association (MD = 8.13; 95% CI: 5.07‒11.19, *P* < 0.001). Regarding gender, 11 studies^5-12,14-16^ evaluated this parameter as a risk factor, reporting a 1.78-times higher probability of AC in men than in women, with statistically significant differences (OR = 1.78; 95% CI: 1.25‒2.53, *P* < 0.01).

Harmful habits (smoking and drinking) were also examined in AC patients compared to the controls without AC. Twelve studies^5-16^ considered that smoking increased the risk of developing AC 1.32 times, with a statistically significant relationship (OR = 1.32; 95% CI: 1.02‒1.71, *P* = 0.04).

**Table 2:** Analysis of main risk factors for actinic cheilitis

**Risk factor**	**References**	**Value**	**OR/MD**	**[95% CI]**	**I** ^2^ **(%)**	***P*** ** value**
Age groups	6,7,11-14,17	>50 years	OR: 3.01	[1.88 to 4.80]	72%	<0.001*
Age	7,12,15,16	mean age	MD: 8.13	[5.07 to 11.19]	76%	<0.001*
Gender	6-17	male	OR: 1.78	[1.25 to 2.53]	76%	<0.01*
Smoking	6-17	yes	OR: 1.32	[1.02 to 1.88]	60%	0.04*
Drinking	6,8,9,11,13,14,17	yes	OR: 1.56	[1.29 to 1.88]	10%	<0.001*
Cumulative sun exposure	7-14	high	OR: 2.13	[1.21 to 3.72]	81%	<0.01*
Daily sun exposure	7-9,12,17	high (≥4 hours)	OR: 2.00	[1.03 to 3.87]	79%	0.04*
Daily sun exposure	7,12,16	mean hours	MD: 9.25	[3.44 to 15.07]	87%	<0.01*
Skin phototype^a^	6-9,11-13,15-17	low (I to III)	OR: 3.30	[2.25 to 4.83]	78%	<0.001*
Sunscreen	8,9,12-14,16,17	yes	OR: 1.03	[0.63 to 1.69]	79%	0.91
Lip balm	8,9,16,17	yes	OR: 1.54	[1.06 to 2.25]	16%	0.02*
Clothes	8,9,12,14,16	cap/hat	OR: 1.06	[0.40 to 2.81]	85%	0.91

OR: Odds Ratio; MD: Mean Difference; CI: confidence Interval; I^2^(%): Higgins statistic for heterogeneity (percentage). ^a^According to Fitzpatrick skin type; *statistically significant.

In seven studies,^5,7,8,10,11,14,15^ alcohol beverage intake was assessed in AC patients and controls, demonstrating that drinkers had 1.56 times more risk of AC. Statistical analysis indicated a highly significant association (OR = 1.54; 95% CI: 1.29‒1.88, *P* < 0.001).

Eight studies^5-7,9,11,14-16^ reviewed the possible implication of accumulated sun exposure during life. AC patients were 2.13 times more likely to have high sun exposure, with highly significant statistical differences (OR = 2.13; 95% CI: 1.21‒3.72, *P* < 0.01). Five studies^6,9,10,14,15^ evaluated daily sun exposure, reporting a two-fold probability of presenting high daily exposure in AC patients (≥4 hours a day). The statistical analysis showed a significant association (OR = 2.00; 95% CI: 1.03‒3.87, *P* = 0.04). Three studies^6,9,13^ investigated the mean number of hours of sun exposure as a possible risk factor, showing, on average, 9.25 more hours of sun exposure in AC patients, with a statistically significant relationship (DM = 9.25; 95% CI: 3.44‒15.07, *P* < 0.01).

Ten studies^5-10,12-15^ analyzed the possible influence of the skin phototype on the AC risk. Subjects with low skin phototypes (I, II, III) exhibited 3.30 times higher AC risk with a highly significant statistical relationship (OR = 3.30; 95% CI: 2.25‒4.83, *P* < 0.001).

Seven studies^5,7,10,11,13-15^ considered the use of sunscreens, finding no relevant impact of their use on the AC risk (OR = 1.03; 95% CI: 0.63‒1.69, *P* = 0.91). Other four studies^10,13-15^ detailed the use of lipstick, verifying that it did not reduce the AC risk. Statistically significant differences were observed (OR = 1.54; 95% CI: 1.06‒2.25, *P* = 0.02). Finally, five studies^6,11,13-15^ examined the use of a cap/hat, reporting no effect on the AC risk. The statistical analysis did not reveal a statistically significant relationship (OR = 1.06; 95% CI: 0.40‒2.81, *P* = 0.91).

## Discussion

Twelve studies on the possible risk factors for AC were included in the present meta-analysis.

AC is a potentially malignant oral disorder characterized by chronic inflammation of the lip, most commonly affecting the lower lip, resulting from excessive and cumulative sun exposure. The rate of malignant transformation of AC to lip squamous cell carcinoma is around 3%.^3^

In the present study, subjects >50 years of age had a 3-fold higher probability of developing AC with highly significant differences (*P* < 0.001). The seven studies^5-11^ that analyzed this parameter coincided in pointing out this higher AC frequency with aging. Older age is a variable strongly associated with the development of AC. The relationship between an increase in age and the presence of AC tends to be directly proportional to the accumulated UV radiation exposure during life, especially in people with fair skin.^10^ Most cases of AC appear in people over 50 years of age who have a longer time of sun exposure and more severe lip clinicopathological changes.^5^ In this investigation, the AC patients had a mean age of 8.13 years older than the controls with a highly significant statistical relationship (*P* < 0.001). All the studies^6,9,12,13^ indicated this higher mean age in AC patients closely associated with a higher mean number of years of exposure to solar radiation.^13^

In the present paper, AC was more frequent in men than in women, with a statistically significant association (*P* < 0.01). Of the eleven studies that considered gender, ten studies^5-7,9-12,14-16^ consistently indicated a predilection for the male gender. At the same time, only one^8^ did not observe this higher prevalence in men, although without statistically significant results. This higher prevalence of AC in men could be explained by their greater exposure to solar radiation derived from professional outdoor activities (farmers, sailors, construction workers, etc.) and by the lack of solar protective measures. All this contributes to the development of chronic lip lesions.^15^ In the case of women, several reasons justify this lower predisposition to AC: a higher frequency of using sunscreen lipsticks and other protective measures, a lower percentage of outdoor workers, or a retirement age lower than that of men.^9^

Harmful habits such as smoking and/or drinking were also analyzed. In this study, smokers had 1.32 times more AC risk with statistically significant differences (*P* = 0.04). Of the 12 studies that evaluated the smoking habit, nine^5-7,9,10,13-16^ confirmed the impact of tobacco consumption, while the remaining three^8,11,12^ noted a higher frequency of AC in non-smokers. The placement of the cigarette on the lip and the continuous exposure to the heat generated by the smoke seem to enhance the lip changes induced by chronic exposure to solar radiation, also increasing the probability of malignant degeneration due to a synergistic action of several carcinogens.^9^ It is important to discourage smoking, especially among people exposed to more than one risk factor involved in lip and oral carcinogenesis.^6^

Similarly, drinking increased the probability of AC 1.56 times, with a highly significant statistical association (*P* < 0.001). The seven studies^5,7,8,10,11,14,15^ that assessed this parameter confirmed the relevance of alcohol intake as a possible causal agent of AC. Although there is no consensus regarding the influence of alcohol on the development of AC due to the difficulty for an adequate quantification of this parameter, alcohol consumption and other factors, especially smoking, favor the development of these labial lesions.^5^ This higher AC prevalence in drinkers might be associated with other risk factors such as more frequent smoking in men, outdoor activities with greater exposure to solar radiation, and the lower use of lip protection factors.^7^

Cumulative sun exposure, both in the number of hours per day and throughout life, is considered the most important etiological agent in the appearance of AC. In this study, greater sun exposure doubled the AC risk with a statistically significant relationship (*P* < 0.01). Six studies^6,7,9,11,14,16^ highlighted the relevance of accumulated sun exposure as a causal agent of AC. Individuals who are regularly sun-exposed at an early age and with a history of accumulated sun exposure during their life are the most susceptible to developing AC.^11^ Likewise, in the present study, patients with high daily sun exposure were twice as likely to present AC with a statistically significant association (*P* = 0.04). The five studies^6,9,10,14,15^ that examined daily hours of sun exposure confirmed this increased AC risk. Sun exposure of more than 4 hours a day is directly associated with an increased AC risk, especially in those who do not use sunscreens.^6^ On the other hand, in this investigation, it was also found that AC patients had a mean number of 9.25 hours more sun exposure than subjects without AC, with statistically significant differences (*P* < 0.01). All the studies^6,9,13^ that estimated the number of hours confirmed this increased AC risk as the number of hours of sun exposure increases. In AC, the tissue damage induced by sun exposure is cumulative, showing the chronicity of the lip lesion.^13^

In the present meta-analysis, people with low skin phototype (I, II, and III) increased the probability of having AC 3.30 times, with a highly significant statistical relationship (*P* < 0.001). The ten studies^5-10,12-15^ that investigated the phototype indicated this increased AC risk in fair-skinned individuals. The lower the skin phototype, the lower the melanin concentration at the level of the basal layer of keratinocytes, the natural pigment with a protective effect against UV radiation.^8^ Besides, subjects with low phototypes have a less effective mechanism for cellular repair of the damage induced by sun exposure.^5^ There is an inverse relationship between the amount of melanin and DNA damage induced by exposure to UV radiation. Furthermore, white-skinned subjects do not present UV-induced apoptosis. In contrast, darker-skinned subjects do show relevant apoptosis, demonstrating the efficient elimination of cells damaged by UV rays in individuals with higher skin phototypes.^12^

In this study, the use of sunscreen creams did not influence the risk of AC, without a statistically significant association (*P* = 0.91). Of the seven studies that assessed this parameter, four^7,10,13,15^ observed AC in the sunscreen users, while the remaining three^6,11,14^ found AC in those who did not use sunscreens. Sunscreens protect the facial skin against solar keratosis; however, since they are not applied to the lips, they do not have any protective effect on them and do not prevent AC in susceptible individuals.^15^

The specific use of lipstick as a protection measure was also verified, surprisingly showing that it did not reduce the AC risk with a statistically significant result (*P* = 0.02). The four studies^10,13-15^ that analyzed this variable confirmed the unexpected ineffectiveness of lipsticks as a protective factor against AC. A possible explanation for this contradictory result could be in the type of population considered in these studies. The majority were rural male workers with little awareness of the use of lipsticks and other protective measures against solar radiation.^14^

Finally, the use of a cap/hat as an element of protection was evaluated, and as with the rest of the protective measures (photoprotective creams and lipsticks), it did not affect the AC risk in a relevant way, without statistically significant differences (*P* = 0.91). Only two^6,11^ of the five studies indicated a protective effect of the cap/hat against AC. On many occasions, the use of a cap/hat creates a false sense of protection when some of these clothes, due to their design, do not cover the lower third of the face, exposing the lips, especially the lower lip, to the action of solar radiation, facilitating the development of AC.^14^

### Limitations of the Study

The results of this meta-analysis should be interpreted with caution due to the high heterogeneity observed in some of the comparisons. The differences in the individual results of the included studies might have conditioned the results of the present study.

It is difficult to evaluate some parameters, such as harmful habits, due to the subjective underestimation carried out by the patients. Furthermore, the precise quantification of the time and intensity of sun exposure is also complicated, even more so when this is the most important etiological factor in this disorder.

The lack of studies with long-term follow-ups of patients with AC makes it difficult to determine the rate of malignant transformation of these lesions to lip cancers.

## Conclusion

In this meta-analysis, the factors from highest to lowest risk of AC were: having a low skin phototype (OR: 3.30), age >50 years (OR: 3.01), having high sun exposure, both cumulative throughout of life (OR: 2.13) and daily (OR: 2.00), being male (OR: 1.78) and being a drinker (OR: 1.56) or smoker (OR: 1.32). However, the use of sunscreen creams and caps/hats to protect against the sun were factors with no significant influence on the AC risk.

## Authors’ Contributions

ARA and AIB contributed equally in concept, design, data collection, and interpretation and preparation of the final manuscript.

## Acknowledgments

None.

## Funding

None.

## Competing Interests

None.

## Ethics Approval

Not applicable.
